# Castleman’s Disease Intra Parotid, a Case Report and Literature Review

**DOI:** 10.30476/DENTJODS.2020.85683.1144

**Published:** 2021-09

**Authors:** Neda Kardouni Khoozestani, Mahdi Niknami, Koroush Ghanbarzadeh, Paniz Ranji

**Affiliations:** 1 Dept. of Oral and Maxillofacial Pathology, School of Dentistry and Cancer Institute, Tehran University of Medical Sciences, Tehran, Iran; 2 Dept. of Oral and Maxillofacial Radiology, School of Dentistry, Tehran University of Medical Sciences, Tehran, Iran; 3 Dept. of Plastic Surgery, Cancer Institute, Tehran University of Medical Science, Tehran, Iran; 4 Resident, Dept. of Oral and Maxillofacial Radiology, School of Dentistry, Tehran University of Medical Sciences, Tehran, Iran

**Keywords:** Castleman’s disease, Intra Parotid, Salivary gland

## Abstract

Castleman’s disease (CD), otherwise known as angiofollicular lymph node hyperplasia, is a rare, poorly understood disorder, which often occurs in a mediastinum. Involvement of parotid gland is a considerably infrequent event. We present a 15-year-old boy patient with a swelling in the left parotid gland that has been diagnosed with CD. The clinical features, radiographic findings, and treatment plan are discussed. Furthermore, a thorough literature review demonstrated 57 published cases of CD in salivary gland with their summarized features.

## Introduction

Castleman’s disease (CD) is a rare non‐neoplastic lymphoproliferative disorder, first known in 1956 by Dr. Benjamin Castleman who described a 40-year-old male with a mediastinal lymph node mass characterized histologically by lymph node hyperplasia and follicles with hyalinized centers [ 1 ]. This disease can occur in any lymphatic chain, and mediastinum is the most common site (60-86%). Salivary gland involvement is greatly rare (6-14%) and very few cases have been reported to date [ 2 ]. Because of unknown etiology, it is represented by various terminologies such as lymphoid hamartoma, giant lymph node hyperplasia, angiofollicular hamartoma, and benign giant lymphoma [ 3 ].

The CD diagnosis is basically confirmed by biopsy. The characteristic histopathological criteria included angiofollicular lymph node hyperplasia in a lymph node region [ 4 ]. Due to the infrequency of CD, the data obtained from literature is more from case series and retrospective studies. In this report, we describe a rare case of unicentric CD presenting in left parotid gland as the first manifestation. Due to an increase risk of the development of lymphoma, complete diagnostic workup including clinical presentation, radiological imaging, and histopathological assessment should be performed very early to arrive at an accurate diagnosis and to provide appropriate therapy.

## Materials and Method

**Figure 1:**
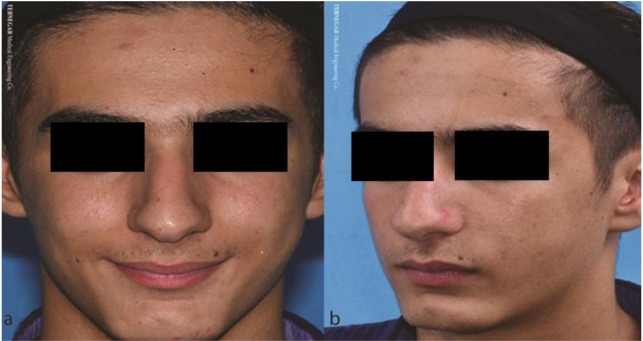
Clinical aspect of the patient showing slight expansion in left parotid gland. a: Frontal, b: Oblique view

**Figure 2:**
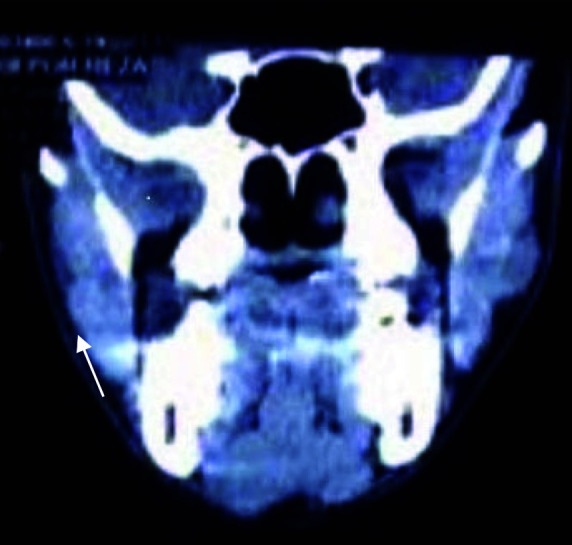
Computed tomography without contrast (coronal view) showing small well-defined hypo dense solid lesion adjacent to the left masseter muscle and in the superficial lobe of left parotid gland

A 15-year-old boy patient referred to Plastic Surgery Department at Imam Khomeini Cancer Institute with chief complaint of left facial swelling for past 4 years (Figure 1a and b). He had history of trauma for 5days. On physical examination, the mass was 1×1cm oval and bean shaped, firm, non-tender with no sign of inflammation, which was snaky in onset and progressed slowly. Paranasal bones encroachment were unremarkable. The parotid duct in the affected side had normal physical appearance without debris or purulent discharge. In the first visit, diagnosis of mucocele was made and aspiration was carried out but it was negative. After one week of follow-up, no changes were observed in mass size and further clinicoradiological evaluation was administered. In the axial and coronal computed tomography (CT) scan slices without contrast, multiple small adenopathy were seen at the both side of neck. There was also a small well-defined hypo dense solid lesion adjacent to the left masseter muscle and in the superficial lobe of left parotid gland. No calcification or necrotic areas were seen within the lesion (Figure 2).

Following the CTs, ultrasonography (US) examination was administrated for the patient and in the US findings; an oval and bean-shaped mass measuring approximately 30*6.5 mm accompanied by centripedal vascularity in the buccal space was reported. In addition, presence of multiple giant lymph nodes with increased wall thickness in the both sides of jugular space and submandibular space was noted. The patient underwent a left superficial parotidectomy with preservation of the facial nerve under general anesthesia. On gross examination tangray salivary gland was identified, which showed central area of well-demarcated gray nodule with firm consistency. Microscopic examination of referred specimen revealed salivary gland tissue composed of mostly serous acini and related ducts with preserved architecture and intra parotid lymph node with marked vascular proliferation and hyalinization of germinal center with appearance corresponded to transform germinal centers regressively. Concentric aggregation of lymphocytes at the mantle zone was noted. Interfollicular stroma showed multiple vessels admixed with few plasma cells. Immuno histo chemistry (IHC) study for CD-21 showed strong positive in follicular dendritic cells within germinal center, which confirmed diagnosis. 

Postoperatively, the patient did well, and he experienced no major complications, that the authors obtained written and signed informed consent from patient's guardians for publishing this case report.

## Discussion

CD is a rare pathological disorder that can rarely affect the parotid gland [ 5 ].Based on analysis of American databases, the incidence of CD is estimated to be 21-25 cases per million person-year [ 6 ]. The most common location of CD is the thorax followed by the neck and abdomen. Within the head and neck region, CD usually presents either as a single nodule under the sternocleidomastoid muscle or as a mass affecting mediastinum to cervical area. Involvement of the salivary gland is considerably infrequent and even more rarely in the parotid gland [ 7 ]. In our literature review, based on available information, parotid was the most common site of involvement compared to other salivary glands. 

In the present case, the lesion was located in buccal side of left parotid gland mimicking a salivary gland tumor. 

 Several theories have been proposed for the un known etiology of this disease. The strongest hypotheses indicate robust relation between virus or chronic inflammation and lymphoproliferation [ 3 ]. In 1970, Flendrig *et al*. [ 8 ] identified distinct histopathologic variants of this lesion and two years later, Keller *et al*. [ 9 ] classified it into the hyaline vascular (HV) and plasma cell types; some patients have a mixed variant.

 Clinically, CD is sub classified into unicentric (UCD) and multicentric (MCD) types. The UCD form usually presents as an asymptomatic painless palpable enlarged lymph node mass which reflects its benign feature. On the other hand, MCD is more aggressive and presents with systemic symptoms such as fever, loss of weight, and splenomegaly and can be associated with some malignancies, such as Kaposi’s sarcoma, non-Hodgkin’s lymphoma, Hodgkin’s disease, and POEMS syndrome (polyneuropathy, organomegaly, endocrinopathy, M protein and skin changes) [ 3 ]. In our review, we found 57 cases; most of them had an asymptomatic swelling similar to the presented case. However, three patients showed symptoms and only one of them suffered from intermittent jaundice.

**Figure 3:**
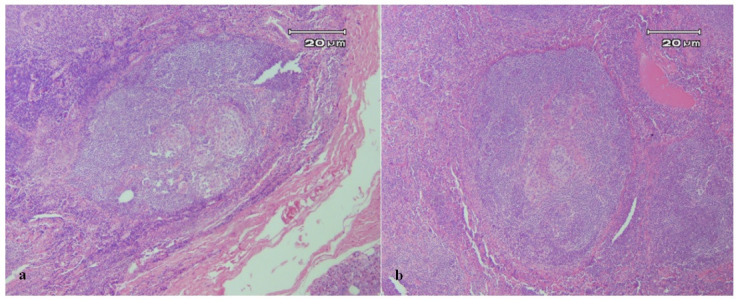
Microscopic images showing hyaline vascular features including, a: two small germinal center giving twining appearance, b: atrophic germinal center and a radially penetrating sclerotic blood vessel. (Lollipop sign)

According to histopathological pattern, the HV is the most common subtype, accounting for 80-90 % of the cases [ 2 ]. This variant occurs over broad age range and, in most studies, males and females were equally affected. HV-CD can be divided into two different groups: follicular and interfollicular region changes. In classical HV-CD, the overall lymph node is preserved but inflected. Lymphoid follicles grow, scatter throughout the cortex and medulla, and commonly consist of two or more small germinal centers (socalled “twinning”) [ 10 ]. In our case, sclerotic blood vessels radially penetrated the germinal centers, which are known as “lollipop lesions” (Figure 3a and b). Twenty two percent of the localized and plurality of MCD is plasma cell type. The mixed type is scarce and characterized by a mixture of the two types [ 2 ]. Of the 58 specimens, 54 were HV (93.1%), two were mixed (3.4%) and two were plasma-cell type (3.4%). Regarding to clinicopathologic correlation, the main differential diagnosis were included toxoplasma lymphadenitis, reactive lymphadenitis and for more severe lesions, follicular lymphoma and mantle cell lymphomas were considered. The formers are excluded by means of large sized follicles, well-formed light and dark zones, tangible body macrophages, and intact sinuses while the latter ones are ruled out by presence monotonous population of neoplastic cell with lack of prominent vascularity in interfollicular region [ 10 ]. A review literature of available published cases for CD revealed more than 50 cases; their features are summarized in Table 1. This information reflects the rare incidence of called disease. Since the disease shows nonspecific clinical, radiological, or cytological features; the diagnosis is challenging and should be made by the aid of histopathological pattern as it was mentioned earlier. Radiographic findings can be helpful for differential diagnosis. CTs with contrast showed a dense enhanced oval, homogenous mass, which reflect hyper vascularity of these lesions. US is also feasible to confirm these features. The prognosis of CD is unpredictable and depends fundamentally on disease subtype [ 11 ].

**Table1:** Summarizes of published case report with Castleman’s Disease in salivary gland

Name	Year	Sex	Age	Location	u/m centric	Histology pattern	Clinical course	Treatment
Cavallaro, *et al*. [ 13 ]	1985	F	23	Parotid	U	HV	Asymptomatic	Complete excision
Prasad H, *et al*. [ 14 ]	1988	M	60	Parotid	U	Plasma cell	Tenderness	Complete excision
Woolgar, *et al*. [ 15 ]	1991	F	24	Parotid	U	HV	Asymptomatic	Superficial parotidectomy
Latz, *et al*. [ 16 ]	1992	M	38	Parotid	U	HV	Asymptomatic	Radiation
Yi, *et al*. [ 17 ]	1995	M	33	Parotid	U	HV	Asymptomatic	Complete excision
Ahuja, *et al*. [ 18 ]	1995	M	50	Parotid	U	HV	Asymptomatic	Complete excision, lymphadenectomy
Yoo, *et al*. [ 19 ]	1995	M	8	SMG	U	HV	Asymptomatic	Complete excision
Leocata, *et al*. [ 20 ]	1996	F	16	Parotid	U	HV	Asymptomatic	Complete excision
Choi, *et al*. [ 21 ]	1997	M	14	Parotid	U	HV	Asymptomatic	Superficial parotidectomy
Goodisson, *et al*. [ 11 ]	1997	M	36	Parotid	U	HV	Asymptomatic	Superficial parotidectomy
Santonja, *et al*. [ 22 ]	1997	F	5	Parotid	U	HV	Asymptomatic	Complete excision
Panayiotides, *et.al*. [ 23 ]	1998	M	30	Parotid	U	HV	Asymptomatic	Complete excision
Parez, *et al*. [ 24 ]	1999	M	9	SMG	U	HV	Asymptomatic	Complete excision
Nahlieli, *et al*. [ 25 ]	2000	F	18	Parotid	U	HV	Asymptomatic	Complete excision
Sanchez-Cuellar, *et al*. [ 26 ]	2001	M	19	Parotid	U	HV	Asymptomatic	Superficial parotidectomy
Mohan, *et al*. [ 27 ]	2003	M	17	Parotid	U	HV	Asymptomatic	Superficial parotidectomy
Samadi *et al*. [ 28 ]	2003	M	6	Parotid	U	HV	Asymptomatic	Complete excision
Kilty, *et al*. [ 29 ]	2005	F	22	Parotid	U	HV	Asymptomatic	Superficial parotidectomy
		M	26		U	HV	Tenderness	Superficial parotidectomy
		F	50		M	Plasma cell	Asymptomatic	Superficial parotidectomy
Dursun, *et al*. [ 30 ]	2006	M	45	Parotid	U	HV	Asymptomatic	Complete excision
Akdogan, *et al*. [ 31 ]	2006	F	52	Bilateral parotid	U	HV	Asymptomatic	Complete excision
Park, *et al*. [ 32 ]	2008	M	9	Parotid	U	HV	Asymptomatic	Superficial parotidectomy
Erdogan, *et al*. [ 33 ]	2008	F	15	Parotid	U	HV	Asymptomatic	Complete excision
Lee, *et al*. [ 34 ]	2009	F	29	Parotid	U	HV	Asymptomatic	Superficial parotidectomy
Gurbuzler, *et al*. [ 3 ]	2010	F	34	Parotid	U	HV	Asymptomatic	Parotidectomy
Zhong, *et al*. [ 35 ]	2010	4M/6F	10 patients between 13-54	Parotid and neck region.	U	HV	Asymptomatic	Complete excision
Lin, *et al*. [ 36 ]	2010	F	18	Parotid	U	HV	Asymptomatic	Complete excision
Erkan, *et al*. [ 37 ]	2011	F	29	Parotid	U	HV	Asymptomatic	Superficial parotidectomy
Mayadağlı, *et al*. [ 38 ]	2012	M	45	Parotid	U	HV	Malaise	Subtotal parotidectomy with radiotherapy
Reece, *et al*. [ 5 ]	2012	M	46	Parotid	U	HV	Asymptomatic	Complete excision
Üstün, *et al*. [ 39 ]	2012	F	52	Parotid	U	HV	Asymptomatic	Superficial parotidectomy
Temirbekov, *et al*.[ 40 ]	2013	F	35	Parotid	U	HV	Asymptomatic	Superficial parotidectomy
Kumar. *et al* [ 41 ]	2014	M	15	Parotid	U	HV	Asymptomatic	Complete excision
Bollig, *et al*. [ 42 ]	2014	M	14	Parotid	U	HV	Asymptomatic	Superficial parotidectomy
Iaconetta, *et al*. [ 12 ]	2014	F	35	Parotid	U	HV	Asymptomatic	Superficial parotidectomy
Delaney, *et al*. [ 7 ]	2015	M	7	Parotid	U	HV	Asymptomatic	Superficial parotidectomy
		F	11	Parotid	U	HV	Asymptomatic	Superficial parotidectomy
Kishori, *et al*. [ 43 ]	2015	M	47	SMG	U	HV	asymptomatic	Complete excision
Abo-Alhassan, *et al*. [ 44 ]	2015	F	29	Parotid	U	HV	Asymptomatic	Complete excision
Shah, *et al*. [ 45 ]	2015	F	22	SMG	U	HV	Asymptomatic	Complete excision
Malzone, *et al*. [ 46 ]	2016	M	44	SMG	U	HV	Asymptomatic	Complete excision
Lin, *et al*.[ 47 ]	2016	F	46	Parotid	U	HV	Asymptomatic	Parotidectomy
Hamilton, *et al*.[ 48 ]	2017	F	12	SMG	U	HV	Asymptomatic	Complete excision
Ekmekci, *et al*. [ 6 ]	2018	M	59	Parotid	M	mixed	Asymptomatic	Superficial parotidectomy
Zhai, *et al*. [ 49 ]	2019	F	62	Parotid	U	mixed	Intermittent jaundice	Complete excision
Batra, *et al*. [ 2 ]	2019	M	35	Parotid	U	HV	Asymptomatic	Superficial parotidectomy
Xiao-Dong, *et al*. [ 50 ]	2020	M	39	Parotid	U	HV	Asymptomatic	Superficial parotidectomy

F: female, SMG: submandibular Gland, U: unicentric, HV: hyaline vascular

Total excision is the golden standard treatment for UCD in the head and neck area. However, the non-operable patients need radiotherapy. Because of aggressive policy of MCD form, it is only cured by palliative treatment [ 12 ]. According to table 1, the treatment of 56 patients consisted of surgical excision. In addition to surgery, adjuvant radiation therapy was used in the treatment of one of the patients [ 38 ]. Moreover, one reported case of CD of the parotid was successfully cured only with radiotherapy [ 16 ]. In our review, the patient underwent therapeutic procedures that came out to be sufficient. However, after follow up, the patient complained about fever, night sweats, and abdominal masses but unfortunately, the patient refused any complementary assessment process.

## Conclusion

It is crucial for the physicians to be aware of this entity for early diagnosis and to provide timely and adequate treatment. Concerning the possibility of malignant transformation, long follow-ups should be regarded.
